# Aortic reservoir characteristics and brain structure in people with type 2 diabetes mellitus; a cross sectional study

**DOI:** 10.1186/s12933-014-0143-6

**Published:** 2014-10-23

**Authors:** Rachel ED Climie, Velandai Srikanth, Richard Beare, Laura J Keith, James Fell, Justin E Davies, James E Sharman

**Affiliations:** Menzies Research Institute Tasmania, University of Tasmania, 17 Liverpool St., Hobart, Tasmania 7000 Australia; Stroke and Ageing Research Group, Monash Medical Centre, Dept. of Medicine, Southern Clinical School, Monash University, Melbourne, Australia; Developmental Imaging, Murdoch Childrens Research Institute, Melbourne, Australia; School of Health Sciences, University of Tasmania, Launceston, Australia; International Centre for Circulatory Health, Imperial College, London, UK

**Keywords:** Type 2 diabetes mellitus, Central hemodynamics, Aortic stiffness, Magnetic resonance imaging, Vascular cognitive impairment

## Abstract

**Background:**

Central hemodynamics help to maintain appropriate cerebral and other end-organ perfusion, and may be altered with ageing and type 2 diabetes mellitus (T2DM). We aimed to determine the associations between central hemodynamics and brain structure at rest and during exercise in people with and without T2DM.

**Methods:**

In a sample of people with T2DM and healthy controls, resting and exercise measures of aortic reservoir characteristics (including excess pressure integral [P_excess_]) and other central hemodynamics (including augmentation index [AIx] and aortic pulse wave velocity [aPWV]) were recorded. Brain volumes (including gray matter volume [GMV] and white matter lesions [WML]) were derived from magnetic resonance imaging (MRI) scans. Multivariable linear regression was used to study the associations of hemodynamic variables with brain structure in the two groups adjusting for age, sex, daytime systolic BP (SBP) and heart rate.

**Results:**

There were 37 T2DM (63 ± 9 years; 47% male) and 37 healthy individuals (52 ± 8 years; 51% male). In T2DM, resting aPWV was inversely associated with GMV (standardized β = −0.47, p = 0.036). In healthy participants, resting P_excess_ was inversely associated with GMV (β = −0.23, p = 0.043) and AIx was associated with WML volume (β = 0.52, p = 0.021). There were no associations between exercise hemodynamics and brain volumes in either group.

**Conclusions:**

Brain atrophy is associated with resting aortic stiffness in T2DM, and resting P_excess_ in healthy individuals. Central vascular mechanisms underlying structural brain changes may differ between healthy individuals and T2DM.

## Background

Type 2 diabetes mellitus (T2DM) is an important vascular risk factor for cognitive impairment. It is associated with brain atrophy [1], infarcts and cerebrovascular lesions (white matter hyperintensity of presumed vascular origin [WML]) [2], potentially leading to cognitive decline and greater risk for dementia. Age-related vascular factors such as hypertension and aortic stiffening are more prevalent in patients with T2DM [3] and may partly explain the associated structural brain abnormalities [4-6]. Aortic stiffening can limit buffering capacity of the large central arteries such that small changes in cardiac stroke volume can result in excessive rises in local pulsatile pressure [7]. These excess pressures may damage peripheral capillary networks [8], which is of relevance to the brain as a high flow organ with low resistance proximal large vessels and an extensive microcirculation. Consequent damage to the neurovascular unit may be a factor underlying the observed brain atrophy in T2DM.

**Figure 1:**
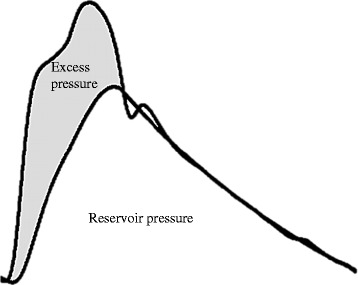
**Example aortic pressure waveform separated into reservoir pressure and excess pressure.** Total measured pressure is equal to the sum of reservoir pressure and excess pressure. Aortic reservoir pressure represents the cyclic increase in aortic volume (aortic distension that occurs during systole) and decrease in volume (aortic recoil that occurs during diastole). Excess pressure is representative of the excess work required by the left ventricle for ejection of stroke volume and is analogous to left ventricular flow.

Aortic reservoir function plays a role in the maintenance of normal central BP and may protect distal microcirculation by dampening excessive aortic pulsatile pressure, as well as reducing peripheral pressure transmission [9]. The aortic reservoir pressure paradigm proposes that the central (aortic) pressure wave may be separated into an aortic reservoir pressure component, representing proximal aortic volume; and an excess pressure (P_excess_) component, representing excess left ventricular work required for stroke volume ejection, analogous to left ventricular flow (Figure 1) [10,11]. Indeed, aortic reservoir pressure is related to aortic stiffness (aortic pulse wave velocity [aPWV]) and we have previously shown that reservoir pressure, not backward pressure (i.e. from peripheral wave reflections) is the largest contributory factor to an increase in augmented pressure [12]. Increased P_excess_ was recently shown to independently predict adverse cardiovascular events in patients with cardiovascular disease [13], possibly due to accelerated target organ damage, but this has never been examined.

Although resting BP indices are clinically important, hemodynamic responses to moderate exercise may have stronger prognostic value in terms of cardiovascular risk [14], suggesting that pathophysiological insight may be gained from exercise hemodynamics beyond that of resting conditions. This may be because individuals can spend a large proportion of their day ambulatory [15] (doing some form of light-moderate physical activity; standing, walking) and the BP response to this type of lower intensity exercise may, therefore, be a better representation of the chronic BP load. Indeed, we have shown that independent of resting BP, light-moderate exercise hemodynamics can unveil BP abnormalities [16,17] and also predict kidney function in older men [18]. We have also found that patients with T2DM have abnormal responses at higher exercise intensities [19,20]. This study aimed to determine associations between central hemodynamics, including aortic reservoir characteristics, and brain structure in people with and without T2DM, during rest and light-moderate exercise.

## Methods

### Study sample

Eighty participants (T2DM n = 40, healthy controls n = 40) were recruited from the community via local advertisements. Exclusion criteria were; pregnancy, arrhythmia, clinical history of cardiovascular disease (including coronary artery disease, myocardial infarction, heart failure or stroke), severe pulmonary disease and contraindication to brain magnetic resonance imaging (MRI). T2DM was determined by self-report of diagnosis by physician. All participants gave informed consent and the study was approved by the University of Tasmania Human Research Ethics Committee.

### Study protocol

Participants attended the testing laboratory on two occasions and were scheduled for MRI assessment. At visit 1, participants were in a post-absorptive state and anthropometric measures, questionnaires relating to BP, medical history and hemodynamic data were recorded. Following 10 minutes of semi-recumbent rest (torso at 45°, arm supported at heart level), brachial BP was measured by a validated automatic device (Omron HEM-907; Hoofddorp, The Netherlands) [21], followed by central hemodynamic variables recorded by applanation tonometry (SphygmoCor, AtCor Medical, Sydney, Australia). All measures were repeated during moderate intensity exercise at 60% of age-predicted maximal heart rate. A validated [22] mercury free sphygmomanometer (UM-101, A&D Medical, Tokyo, Japan) and auscultation was used to measure exercise brachial BP. Details of the exercise protocol have been described elsewhere [18]. Non-invasive impedance cardiography was continuously recorded throughout the protocol (PhysioFlow; Manatec Biomedical; Macheren, France). At visit 2, fasting venous bloods were taken and participants were fitted with a 24-hour ambulatory BP monitor (24ABPM; TM-2430, A&D Medical, Sydney, Australia). Hypertension was defined as clinic brachial BP ≥140/90 mmHg, self-reported diagnosis by a physician, or use of antihypertensive medications.

### MRI analysis

Scans were performed on a 1.5 T General Electric Signa Excite T scanner with the following sequences: High-resolution T1 weighted spoiled gradient echo (TR 35 ms, TE 7 ms, flip angle 35°, field of view 24 cm, voxel size 1 mm^3^) comprising 120 contiguous slices; T2 weighted fast spin echo (TR 4300 ms; TE 120 ms; NEX 1; turbo factor 48; voxel size 0.90 × 0.90 × 3 mm); FLAIR (TR = 8802 ms, TE = 130 ms, TI = 2200 ms, voxel size 0.50 × 0.50 × 3 mm). Scans were registered to a 152 brain Montreal Neurological Institute template in stereotaxic coordinate space. Brain tissue was classified as gray or white matter using statistical parametric mapping software SPM5. Hippocampi were manually segmented using standard landmarks with high test-retest reliability [23]. WML were segmented using a validated semi-automated method [24]. Gray matter (GMV), white matter, WML, and hippocampal volumes were calculated using standard voxel counting algorithms. MRI examiners were blinded to outcome variables and diabetes status.

### Central hemodynamic measures

#### Aortic reservoir characteristics

Central (aortic) pressure waveforms were reconstructed as previously described [25]. Using custom MatLab software the averaged radial pressure waveforms were separated into reservoir pressure (representative of the cyclic changes in aortic volume that occur during systolic expansion to store blood, and diastolic recoil to allow for the discharge of blood from the proximal aorta) and excess pressure (excess work done by the left ventricle, see Figure 1) [10,11]. Reservoir pressure was calculated as previously described [13] and P_excess_ was determined by subtracting the reservoir pressure from the aortic pressure waveform [26].

#### Central BP and aortic stiffness

Central BP was measured in duplicate and augmentation index (AIx), augmented pressure (AP), pulse pressure (PP) and PP amplification were calculated [25]. Right sided carotid-to-femoral aPWV was measured as previously described [18].

#### Cardiothoracic bioimpedance

Measures of cardiac output, systemic vascular resistance, heart rate and stroke volume were recorded using a device with good reproducibility during rest and exercise [27]. Five minutes of continuous steady state monitoring was averaged and analyzed offline.

### Peripheral hemodynamics

Duplicate conventional brachial BP measures were averaged for analysis. 24ABPM was measured every 20 minutes during the daytime, and every 30 minutes during the nighttime.

### Biochemistry

Fasting blood glucose, insulin, glycated hemoglobin (HbA1c), and lipid profiles were obtained by accredited laboratory techniques (Royal Hobart Hospital pathology department). A resting urine sample was analyzed for the presence of albumin by the Royal Hobart Hospital pathology department.

### Statistical analysis

Data were analyzed using SPSS for Windows software version 19.0 (IBM SPSS Statistics, New York, USA). Data were visually inspected for normality of distribution and were all normally distributed. All brain volume outcome measures were expressed as a ratio of total intracranial volume. To compare characteristics between patients with T2DM and healthy participants, independent t*-*tests (continuous variables) and Chi square tests (dichotomous variables) were performed. Independent t*-*tests were used to compare unadjusted brain volumes between groups, followed by analysis of covariance (ANCOVA) adjusted for age and sex. To assess the relationships between resting and exercising central hemodynamic variables and brain volumes, Pearson’s correlations and multivariable linear regression were performed. Z statistic scores were determined to compare the regression slopes obtained from within-group correlations. Independent variables known (age and sex) or suspected (heart rate, ambulatory daytime systolic BP [SBP], body mass index [BMI] and total cholesterol) to contribute to variance in brain volumes were added separately into the regression model, and a p < 0.05 was considered statistically significant. Based on previous reproducibility work [28], we calculated that a between-group difference of 10 mmHg in central SBP could be detected in 36 participants per group (α = 0.05 and β = 0.20), therefore we recruited 40 participants for each group.

## Results

### Sample characteristics

One patient with T2DM and two healthy participants withdrew consent for MRI due to claustrophobia. Brain volume data was unavailable for one patient with T2DM (due to a significant non-vascular abnormality on MRI) and technical difficulty rendered aortic reservoir data unavailable in two participants (one participant from each group), resulting in 37 participants in each group. Compared with healthy participants, patients with T2DM were older, heavier, and had greater blood glucose and (HbA1C). None of the healthy participants were on BP or cholesterol lowering medications (Table 1).

**Table 1:** **Study participant characteristics**

**Variables**	**T2DM (n = 37)**	**Healthy (n = 37)**	**P value**
Male, n (%)	17 (47)	19 (51)	0.56
Age (years)	63 ± 9	52 ± 8	<0.001
Body mass index (kg/m^2^)	30.5 ± 4.8	25.9 ± 3.3	<0.001
Waist:hip (ratio)	0.91 ± 0.06	0.84 ± 0.1	0.002
Current smoker, n (%)	3 (8)	3 (8)	0.97
Hypercholesterolemia, n (%)	25 (66)	10 (27)	0.001
Normotensive, n (%)	15 (39)	28 (76)	0.002
24ABPM systolic BP (mmHg)	134 ± 13	130 ± 11	0.21
24ABPM diastolic BP (mmHg)	75 ± 8	79 ± 6	0.016
Daytime systolic BP (mmHg)	138 ± 14	136 ± 13	0.50
Nighttime systolic BP (mmHg)	119 ± 12	113 ± 11	0.016
Duration of diabetes (years)	6 ± 6	-	-
Antihypertensive medications, n (%)	24 (63)	0 (0)	<0.001
Oral hypoglycemic medications, n (%)	26 (68)	0 (0)	<0.001
Urinary albumin (mg/L)	9.00 ± 11.19	7.85 ± 7.59	0.60
Insulin, n (%)	5 (13)	0 (0)	0.016
Statin, n (%)	25 (66)	0 (0)	<0.001
Glucose (mmol/L)	7.5 ± 1.8	4.7 ± 0.4	<0.001
Glycated hemoglobin (HbA1C) (%)	7.2 ± 0.8	5.5 ± 0.3	<0.001
Insulin (IU/mL)	10.2 ± 8.6	2.4 ± 4.7	<0.001
Total cholesterol (mmol/L)	4.4 ± 1.0	5.4 ± 1.0	<0.001
HDL cholesterol (mmol/L)	1.3 ± 0.4	1.6 ± 0.4	0.002
Triglycerides (mmol/L)	1.4 ± 0.6	1.0 ± 0.5	0.003

Data expressed as mean ± standard deviation or %. T2DM, type 2 diabetes mellitus; 24ABPM, 24 hour ambulatory blood pressure monitoring; BP, blood pressure; HDL, high density lipoprotein. P is for between group analyses.

Table 2 summarizes the difference in brain volumes between groups whilst Table 3 summarizes the differences in resting and exercising central hemodynamics. There was no difference between the groups in any of the adjusted brain volumes. Those with T2DM had significantly greater values in most aortic reservoir characteristics and other hemodynamic variables at rest and during exercise. No between-group differences were observed for reservoir pressure integral and stroke volume during rest and exercise, and for peak reservoir pressure and cardiac output during exercise alone. Compared with healthy participants, brachial SBP and PP were significantly higher in those with T2DM at rest and during exercise, whereas resting systemic vascular resistance was significantly lower. P_excess_ correlated with AP at rest in patients with T2DM (r = 0.49, p = 0.001) and in healthy participants at rest and during exercise (r = 0.58, p < 0.001 and r = 0.34, p = 0.032 respectively). In patients with T2DM, there was a significantly greater change from rest to exercise in peak excess pressure, central SBP, central PP, aPWV, brachial SBP and brachial PP compared to healthy participants (p < 0.05 for all).

**Table 2:** **Brain magnetic resonance imaging (MRI) volumes in patients with type 2 diabetes mellitus (T2DM) and healthy participants**

**MRI variable**	**T2DM**	**Healthy**	**Association of T2DM with MRI variable**	**P for regression**
	**Mean ± SD (n = 37)**	**Mean ± SD (n = 37)**	**β coefficient (95% CI)**	
Gray matter volume (ml)	567.36 ± 77.81	607.81 ± 63.01	0.014 (−17.23, 21.21)	0.84
White matter volume (ml)	583.92 ± 76.03	604.84 ± 80.53	−0.005 (−15.98, 14.48)	0.92
Left hippocampal volume (ml)	2.43 ± 0.37	2.55 ± 0.38	−0.019 (−0.17, 0.14)	0.86
Right hippocampal volume (ml)	2.51 ± 0.36	2.56 ± 0.39	0.046 (−0.14, 0.21)	0.70
White matter lesion volume (ml)	3.34 ± 2.38	3.44 ± 2.39	−0.148 (−1.93, 0.54)	0.26

Unadjusted MRI volumes are presented in the first two columns; β refers to standardized beta coefficient for the association between T2DM and MRI variables determined by ANCOVA and adjusted for age, sex and total intracranial volume. P value is for relation of diabetes status with MRI variables.

**Table 3:** **Differences in central and peripheral hemodynamic variables between patients with type 2 diabetes mellitus (T2DM) and healthy participants at rest, during exercise and the change from rest to exercise**

	**Rest**			**Exercise**			**Change from rest to exercise**		
	**T2DM (n = 37)**	**Healthy (n = 37)**	**P value**	**T2DM (n = 37)**	**Healthy (n = 37)**	**P value**	**T2DM (n = 37)**	**Healthy (n = 37)**	**P value**
**Central hemodynamics**									
Peak reservoir pressure (mm Hg)	36 ± 8	32 ± 4	0.016	18 ± 10	15 ± 5	0.17	−19 ± 10	−17 ± 10	0.49
Reservoir pressure integral (Pa.s)	1872 ± 520	1869 ± 369	0.97	794 ± 485	694 ± 263	0.40	−1413 ± 570	−1441 ± 454	0.81
Peak excess pressure (mm Hg)	35 ± 9	30 ± 4	0.005	73 ± 16	58 ± 12	<0.001	37 ± 19	27 ± 11	0.013
Excess pressure integral (Pa.s)	630 ± 197	493 ± 98	<0.001	1644 ± 437	1255 ± 472	<0.001	970 ± 468	776 ± 470	0.079
Central systolic BP (mm Hg)	114 ± 11	103 ± 10	<0.001	132 ± 14	114 ± 13	<0.001	18 ± 12	11 ± 11	0.015
Central pulse pressure (mm Hg)	45 ± 9	37 ± 5	<0.001	52 ± 12	39 ± 7	<0.001	8 ± 9	2 ± 7	0.003
0.Pulse pressure amplification (ratio)	1.2 ± 0.1	1.3 ± 0.1	<0.001	1.5 ± 0.1	1.6 ± 0.1	0.007	0.26 ± 0.11	0.23 ± 0.09	0.37
Augmented pressure (mm Hg)	13 ± 4.8	8 ± 5	<0.001	9 ± 6	4 ± 4	<0.001	−4 ± 5	−4 ± 3	0.78
Augmentation index (%)	29 ± 6.8	21 ± 10	<0.001	17 ± 9	10 ± 6	0.001	−12 ± 7	−11 ± 6	0.68
Augmentation index (at 75 bpm)	23 ± 6	13 ± 11	<0.001	25 ± 9	14 ± 11	<0.001	0.8 ± 9	1 ± 7	0.73
*Adjusted augmentation index (%)	26 ± 6.7	23 ± 6.7	<0.001	14.6 ± 8.0	11.9 ± 7.9	<0.001	−11.6 ± 6.7	−11.5 ± 6.8	0.58
Aortic pulse wave velocity (m/s)	8.01 ± 2.16	6.29 ± 1.42	<0.001	9.73 ± 2.10	7.02 ± 1.43	<0.001	2.14 ± 2.59	0.32 ± 2.71	0.004
Heart rate (bpm)	64 ± 8	58 ± 8	0.001	92 ± 12	86 ± 12	0.043	27 ± 9	28 ± 9	0.58
Cardiac output (L/min)	5.24 ± 0.90	4.50 ± 0.73	<0.001	8.22 ± 1.54	7.91 ± 1.28	0.35	2.9 ± 1.3	3.4 ± 1.3	0.103
Stroke volume (mL)	82 ± 11	78 ± 15	0.26	90 ± 13	93 ± 14	0.36	7 ± 12	14 ± 11	0.017
**Peripheral hemodynamics**									
Brachial systolic BP (mm Hg)	124 ± 12	114 ± 9	<0.001	155 ± 17	134 ± 14	<0.001	31 ± 13	20 ± 13	<0.001
Brachial diastolic BP (mm Hg)	68 ± 8	65 ± 6	0.064	77 ± 9	73 ± 9	0.097	8 ± 6	9 ± 8	0.93
Brachial pulse pressure (mm Hg)	55 ± 10	49 ± 5	0.002	78 ± 15	60 ± 10	<0.001	23 ± 11	11 ± 10	<0.001
Systemic vascular resistance (d.s.cm^−5^)	1369 ± 243	1503 ± 268	0.027	1004.38 ± 201	973 ± 157	0.45	−354 ± 206	−529 ± 247	0.001

Data expressed as mean ± standard deviation. BP, blood pressure. P is for between group analyses. *Augmentation index adjusted for age, sex, heart rate and height.

### Associations between central hemodynamic and brain MRI volumes

**Figure 2:**
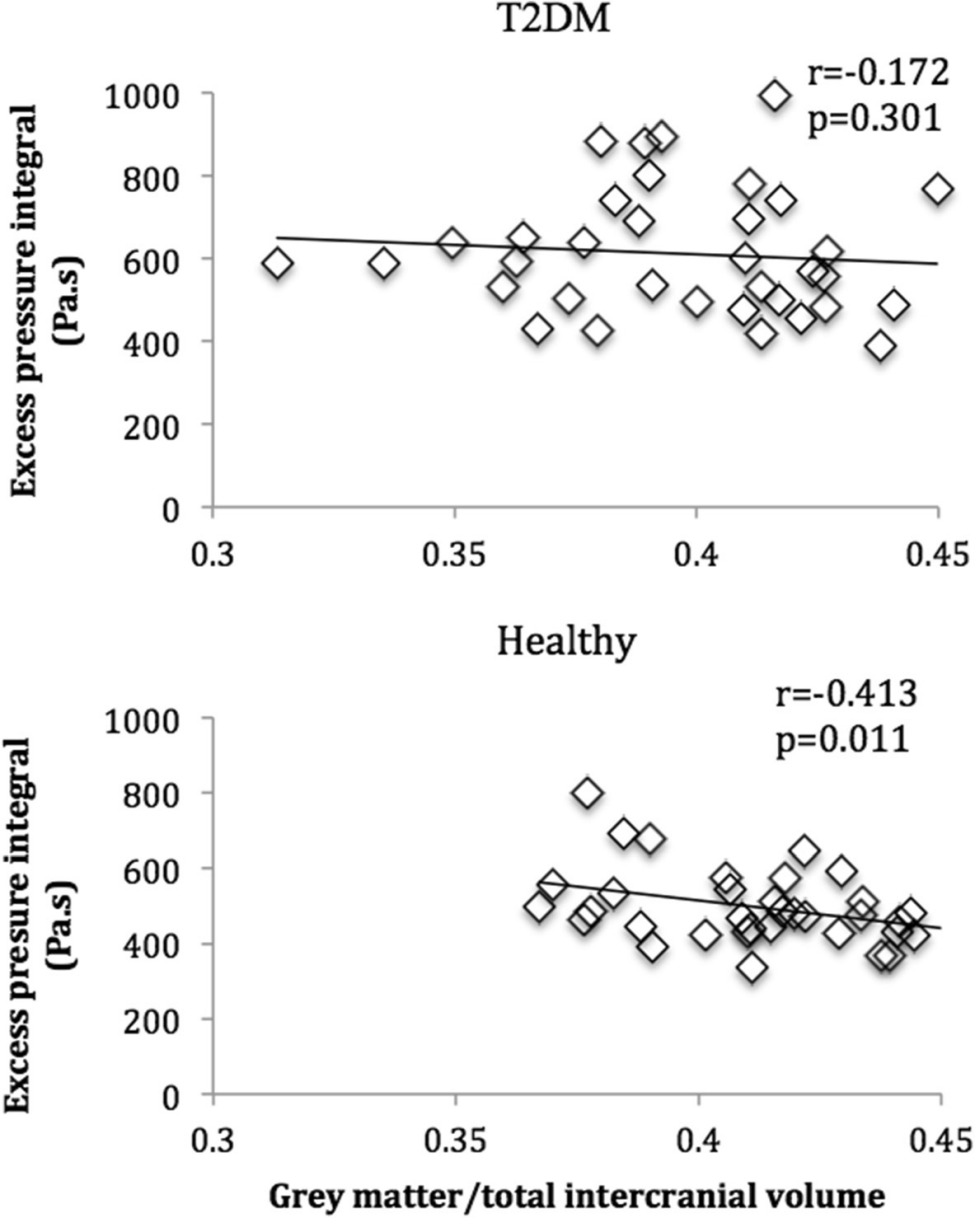
**Univariate association (unadjusted) between excess pressure integral and gray matter volume in patients with type 2 diabetes mellitus (T2DM) and healthy participants at rest.**

In patients with T2DM, resting aortic reservoir characteristics were not related to MRI volumes (p > 0.05 for all). In healthy participants, there was an inverse correlation between resting P_excess_ and GMV (r = −0.41, p = 0.011), which remained after adjusting for age, sex, ambulatory daytime SBP and heart rate (Table 4, Figure 2). Further adjustment for BMI or urinary albumin did not attenuate the association (β = −0.73^−4^, p = 0.028, β = −0.061^−3^, p = 0.045 respectively) however, the addition of total cholesterol did (β = −0.58^−4^, p = 0.060). Adjusting for clinic SBP (in the place of ambulatory daytime SBP), did not affect the relationship between P_excess_ and GMV (β = 0.075^−3^, 95% CI −0.139^−3^ to −0.011^−3^, p = 0.023). There was a between-group difference in the strength of the association between resting P_excess_ and GMV in patients with T2DM compared to healthy participants (z = 2.08, p = 0.044, Figure 2).

**Table 4:** **Multivariable analysis of resting hemodynamics and gray matter volume in patients with type 2 diabetes mellitus (T2DM) and healthy participants**

**Brain MRI variable**	**Independent variable**	**β unstandardized (95% CI)**	**β standardized**	**P value**	**Model adjusted R** ^**2**^
**T2DM**					
Gray matter/total intracranial volume	Aortic pulse wave velocity	−0.007 (−0.014, −0.050^−2^)	−0.47	0.036	0.16
	Age	−0.001 (−0.002, 0.001)	−0.15	0.44	
	Sex	0.006 (−0.019, −0.030)	0.088	0.64	
	24ABPM daytime systolic BP	2.94^−5^ (−0.001, 0.001)	0.013	0.94	
	Heart rate	0.001 (−0.001, 0.001)	0.18	0.28	
**Healthy**					
Gray matter/total intracranial volume	Excess pressure integral	0.60^−4^ (−0.119^−3^, −0.200^−5^)	−0.23	0.043	0.68
	Age	−0.020 (−0.002, −0.001)	−0.49	<0.001	
	Sex	−0.028 (−0.039, −0.018)	−0.55	<0.001	
	24ABPM daytime systolic BP	4.30^−5^ (−0.390^−3^, 0.477^−3^)	0.021	0.84	
	Heart rate	0.32^−4^ (−0.001, 0.001)	−0.010	0.93	
White matter lesion/total intracranial volume	Augmentation index	5.91^−5^ (0.9^−5^, 0.12^−3^)	0.52	0.021	0.16
	Age	2.01^−5^ (0.29^−4^, 0.7^−4^)	0.14	0.41	
	Sex	0.28^−3^ (−0.001, 0.001)	0.12	0.57	
	24ABPM daytime systolic BP	−0.10^−4^ (−0.39^−4^, 0.22^−4^)	−0.093	0.58	
	Heart rate	9.17^−6^ (−0.41^−4^, 0.59^−4^)	0.060	0.71	
	Central pulse pressure	0.11^−3^ (0.28^−3^, 0.19^−3^)	0.48	0.010	0.19
	Age	2.79^−5^ (−0.18^−4^, 0.74^−4^)	0.19	0.23	
	Sex	0.12^−3^ (−0.001, 0.001)	−0.045	0.79	
	24ABPM daytime systolic BP	0.17^−4^ (−0.49^−4^, 0.14^−4^)	−0.093	0.58	
	Heart rate	1.40^−5^ (−0.35^−4^, 0.63^−4^)	0.091	0.57	

R^2^ refers ANOVA adjusted R square and P value is for the independent variable. 24ABPM, 24 hour ambulatory blood pressure monitoring; BP, blood pressure. All models adjusted for age, sex, ambulatory daytime systolic BP and heart rate.

In patients with T2DM, but not in healthy participants, resting aPWV was inversely associated with GMV (r = −0.45, p = 0.005) and remained associated after adjusting for age, sex, ambulatory daytime SBP, heart rate (Table 4) and the use of antihypertensive medication. Additionally, adjusting for clinic SBP instead of ambulatory BP, did not affect the relationship between aPWV and GMV (β = −0.009, 95% CI −0.015 to −0.002, p = 0.009). Further adjustment for BMI, urinary albumin or total cholesterol did not alter the association (β = −0.007, p = 0.036, β = −0.007, p = 0.050 and β = −0.006, p = 0.045 respectively). There was no difference between healthy participants and patients with T2DM in the strength of the association between aPWV and GMV (z = 1.76, p = 0.088).

Aortic reservoir characteristics were not related to WML volume in either group (p > 0.05 for all). In healthy participants, resting AIx and central PP were the only hemodynamic variables associated with WML volume (r = 0.46, p = 0.004 and r = 0.47, p = 0.003 respectively) and remained related after adjusting for age, sex, ambulatory daytime SBP and heart rate (Table 4). Alternatively adjusting for clinic SBP, in the place of ambulatory BP, did not attenuate the relationships between central PP and AIx with WML (β =0.122^−3^, 95% CI 0.013^−3^ to 0.230^−3^, p = 0.029 and β =8.354^−5^, 95% CI −0.018^−3^ to −0.149^−3^, p = 0.014 respectively). Further adjustment for BMI, urinary albumin or total cholesterol did not attenuate the association between AIx and WML volume (β = 5.40^−5^, p = 0.037, β = 6.233^−5^, p = 0.020 and β = 5.86^−5^, p = 0.025) or central PP and WML volume (β = 9.83^−5^, p = 0.025, β = 0.120^−3^, p = 0.007 and β = 0.0001, p = 0.006 respectively). Neither exercise central hemodynamic variables nor the peripheral hemodynamic variables were associated with MRI brain volumes in either group.

## Discussion

To our knowledge, this is the first study to examine associations between aortic reservoir characteristics and brain structure. There are several new or noteworthy findings: 1) In healthy individuals, P_excess_ (a novel marker of cardiovascular risk) was independently associated with GMV. 2) In patients with T2DM, aortic stiffness (a more traditional marker of cardiovascular risk and shown to be elevated in patients with T2DM) was independently associated with GMV. 3) Contrary to expectation, exercise hemodynamic variables were not stronger correlates of brain structural abnormalities than resting variables. Overall, these findings suggest that central hemodynamic mechanisms may play a role in leading to structural brain changes underlying cognitive impairment, but that these mechanisms may differ between healthy individuals and patients with T2DM.

Unique to the brain is the continuous passive perfusion of high volume blood flow to the organ throughout systole and diastole [29]. High flow associated with low microvascular resistance could lead to brain vascular networks being sensitive to upstream changes in pressure and flow pulsatility [30,31]. Maintenance of relatively low central BP (especially PP) could, therefore, be important in protecting the microcirculation from excess pressure and/or flow pulsatile energy which may lead to microvascular remodeling, ischemia or structural brain changes [30]. This hypothesis appears to be consistent with data in our study showing an independent association of WML (a marker of small cerebral vessel disease) with raised central PP and AIx in healthy people. Moreover, we show that higher P_excess_ is related to lower GMV in this population. P_excess_ is representative of the excess left ventricular work required above the minimum to eject blood into the aorta and the P_excess_ waveform has been shown to correspond closely with the flow velocity waveform [10,11,13,26]. Thus one interpretation of the association between high P_excess_ and low GMV is that greater pressure and/or flow transmission from the aorta to the cerebral circulation causes microvascular stress [5], unfavorable remodeling leading to ischemia [30] and neuronal loss.

Despite patients with T2DM being significantly older, of greater BMI and aortic stiffness compared to controls, there were no significant differences between the groups in any of the brain volume measures. This may be explained by the relatively small sample size or by the relatively younger age and shorter duration of T2DM than that of previous studies showing a significant reduction in brain volume compared to non-diabetic individuals [32,33]. On the other hand WML volume has been shown to not differ between patients with T2DM and age and sex matched controls [34]. Interestingly, the relationship between high P_excess_ and low GMV was only evident in healthy individuals, whereas adverse structural brain changes were more highly related to aortic stiffness in patients with T2DM. These results may be influenced by the cross sectional design of the study, but it is also likely that alterations in central hemodynamic function associated with T2DM is an explanatory factor. Key differences in patients with T2DM compared with healthy individuals were increased aortic stiffness, higher cardiac output (mainly due to higher heart rate) and reduced systemic vascular resistance. Increased aortic stiffening has previously been described in these patients, and other study samples have observed similar high left ventricular flow output [35], reduced peripheral resistance and different central hemodynamic responses to postural stress [25,36]. The association between aortic stiffness and brain structural defects has not been definitively established in patients with T2DM despite some studies showing evidence for [37], however also against [38], an association with cognitive impairment. Our findings agree with data from patients with type 1 diabetes mellitus [39] and the general community in which aortic stiffening was independently related to brain structural defects [30,31].

We can only speculate as to possible mechanistic differences explaining brain atrophy between healthy participants and those with T2DM. During systole, pressure rises due to increased aortic inflow relative to outflow [11]. A proportion of the pressure rise is dispersed via aortic reservoir function which is dependent on proximal aortic stiffness and peripheral resistance, both aiding in buffering BP fluctuations to allow steady blood flow to the periphery. Aortic reservoir pressure integral was not different in those with T2DM compared with healthy controls despite higher cardiac output and increased aortic stiffness in the former. This is similar to previous reports whereby patients with T2DM were shown to have reduced aortic elastic properties, however, there was no difference in aortic energy loss compared to non-diabetic controls [40]. This implies that the significant reduction in systemic vascular resistance in patients with T2DM may be a factor mitigating excessive increases in aortic reservoir pressure. Alternatively, or in conjunction, despite some studies showing smaller aortic root diameter in patients with T2DM [41], aortic diameter could have remodeled to be higher in patients with T2DM in the current study, thereby enabling relatively more inflow into the proximal aorta before a rise in pressure occurs. Others have suggested that alterations in aortic, rather than carotid arterial properties occur in patients with T2DM [42,43]. Impedance mismatching between the aortic and carotid arteries have previously been associated with increased flow pulsatility in the carotid vasculature and may relate to cerebral microvascular remodeling and lower brain volumes [30]. Similarly, our data supports the probability that brain structural defects associated with aortic stiffness in patients with T2DM may be the product of excessive transmission of flow (rather than pressure) pulsatility to the cerebral circulation. Therapeutic methods (such as weight loss and reductions in insulin) that target aortic stiffness [44] may, therefore, be beneficial in patients with T2DM.

Finally, and in opposition to our hypothesis, associations between exercise aortic reservoir characteristics and brain atrophy/WML were not enhanced compared to resting data, despite patients with T2DM having exaggerated hemodynamic responses indicative of central systolic stress (including increased central PP, AIx and aPWV) compared to healthy individuals. This was based on the expectation that moderate exercise (similar to ambulatory BP conditions) would be more representative of the chronic hemodynamic loading experienced during normal daily activity and, thus, would be more highly related to end organ disease. This appears to be relevant to cardiac structure [45] and kidney function [18] but the lack of relationship with brain morphology implies different pathophysiological pathways.

The strengths of our study include comprehensive MRI measures and rigorous hemodynamic examination at rest and during moderate intensity exercise. Despite finding significant associations between central hemodynamic variables and GMV in both patients with T2DM and healthy participants, we have performed multiple statistical tests in a relatively small study sample and, therefore, further studies in larger samples are required to confirm our results. We did not measure aortic root diameter and, therefore, our assumption of aortic dilation cannot be confirmed. Furthermore, the cross sectional nature of the study limits inference regarding causality.

## Conclusions

In summary, this is the first study to examine associations between aortic reservoir characteristics and brain structure. Our findings suggest that P_excess_ may be an important contributor to brain atrophy in healthily ageing individuals whereas in patients with T2DM, aortic stiffening may play a more prominent role. These findings suggest that there may be different vascular abnormalities contributing to brain dysfunction among diabetics compared with non-diabetics. However more work is required to determine the underlying central vascular mechanism/s.
